# Cervical myelopathy due to a solitary osteochondroma: a case report

**DOI:** 10.1186/s40064-016-2183-8

**Published:** 2016-04-27

**Authors:** Toru Asari, Naoki Echigoya, Norihiro Sasaki, Gentaro Kumagai, Kazumasa Ueyama

**Affiliations:** Department of Orthopaedic Surgery, Hirosaki Memorial Hospital, 59-1 Sakaizeki-nishida, Hirosaki, Aomori 036-8076 Japan

**Keywords:** Osteochondroma, Exostoses, Cervical myelopathy, Surgical treatment

## Abstract

**Introduction:**

Osteochondroma is the most common benign bone tumor. However, the incidence of osteochondroma in the spine is reported to be very rare.

**Case description:**

This report presents the case of a 57-year-old man who suffered from osteochondroma of the cervical spine. He had bilateral lower extremity pain for 3 years, developing pain of right upper extremity and gait disturbance. Plain radiographic images and computed tomography scans showed bony lesion in right C6/7 foramen and C6 lamina. Magnetic resonance images of whole spine showed severe compression of spinal cord at the C6/7 and spinal canal stenosis at the L3/4 level. First, we performed a surgery of the cervical spine, and removed the tumor covered with the cartilaginous cap. The pathological diagnosis of the tumor was osteochodroma. After the surgery, the symptoms on his right upper extremity improved smoothly. Because the bilateral lower extremity pain remained, a L3/4 partial laminectomy was performed 1 month later, and the symptom improved. At 1 year after his primary operation, we could not find a recurrence of the tumor.

**Conclusions:**

It is very important to perform a complete *en bloc* resection of the tumor (especially cartilaginous cap) to prevent the recurrence.

## Introduction

Osteochondroma is frequently located in the metaphysis of the long bones, and it is an ectopic development of cartilage growth plate. This tumor constitutes 10–15 % of all bone tumors and 20–50 % of benign bone tumors (Murphey et al. 2000). It typically occurs in young adolescent patients, because it is a disease of bone growth (Brastianos et al. 2005). We frequently treat this tumor in the proximal humerus, distal femur and metaphysis of tibia. However, the incidence rate of the tumor in the spine is very rare, only approximately 3 % (Khosla et al. 1999). In this report, we demonstrate a case of cervical myelopathy due to this rare tumor with a review of the literature.

## Case report

A 57-year-old man had suffered from low back and bilateral lower extremity pain for 3 years, and had been diagnosed with lumbar disc herniation by a general practitioner. He had a history of hyperlipemia and diabetes. However his symptoms were getting worse, and he developed additional symptoms: right upper extremity pain, and gait disturbance. He went to a nearby orthopedic clinic, and was soon referred to us for additional examination and treatment. His neurological examination revealed numbness in the right forearm and both thighs. Weakness of the interosseous muscle on the right side was detected, with grade of 4/5 recorded by manual muscle testing. His gait was very unstable, and he needed to be assisted by a walker. His right patellar tendon and Achilles tendon reflexes were hyperactive and the Hoffmann and Babinski reflex were positive. Laboratory values including full blood count, electrolytes, and inflammatory markers were all within normal limits.

**Fig. 1:**
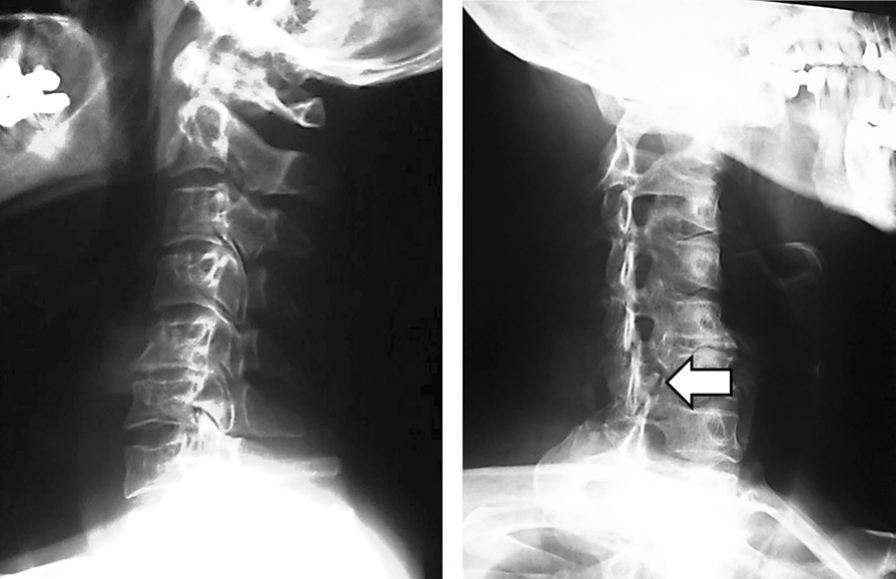
Preoperative plain radiograph of the cervical spine. An osseous protrusion in the right C6/7 foramen could be observed

**Fig. 2:**
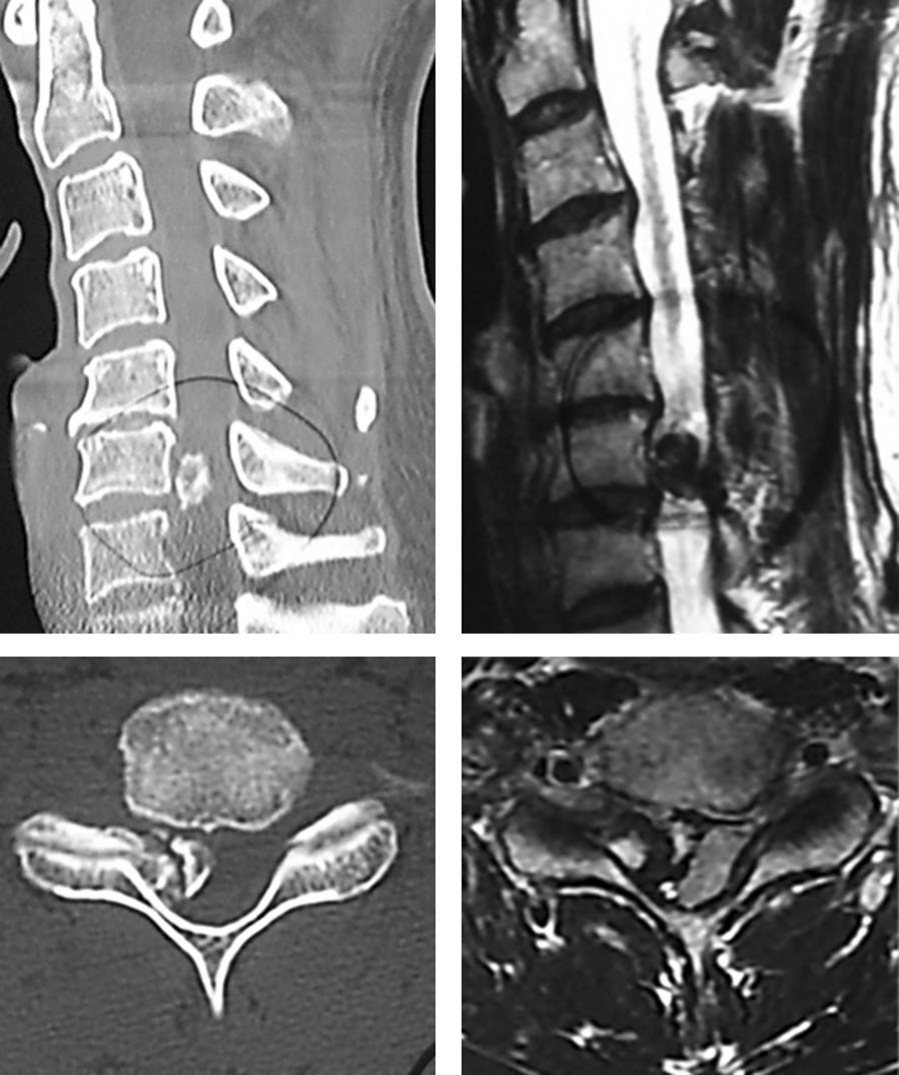
Preoperative CT and MRI of the cervical spine. Sagittal and axial CT scans demonstrated an osseous tumor originating in the neighborhood of the right C6/7 facet joint. Sagittal and axial MRI revealed an extradural mass which compressed the spinal cord severely at C6/7 level

The plain radiological findings of his cervical spine indicated an osseous protrusion in the right C6/7 foramen (Fig. 1). Computed tomography (CT) scans showed an osseous tumor originating in the neighborhood of the right C6/7 facet joint and occupying a part of the spinal canal (Fig. 2). Magnetic resonance images (MRI) revealed the compression of spinal cord at the C6/7 and spinal canal stenosis at the L3/4 level. The tumor was hypointense or isointense on T1-weighted and T2-weighted images.

**Fig. 3:**
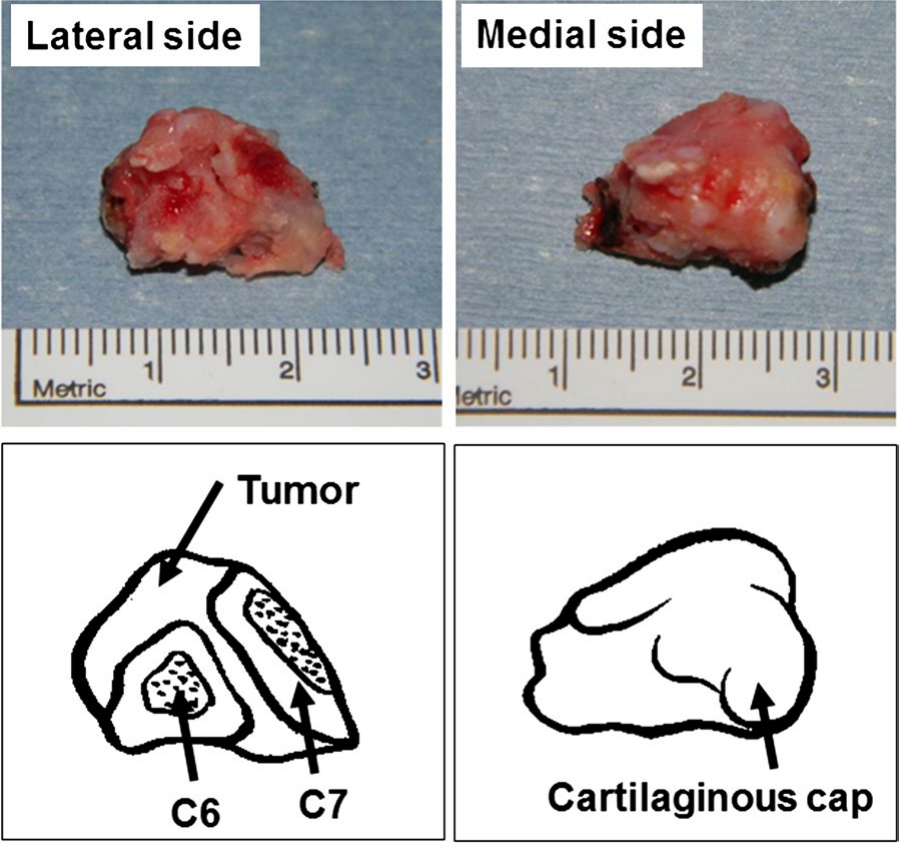
Operative specimen was covered with cartilaginous tissue. The tumor originated from the neighborhood of the right C6/7 facet joint and occupied part of the spinal canal

**Fig. 4:**
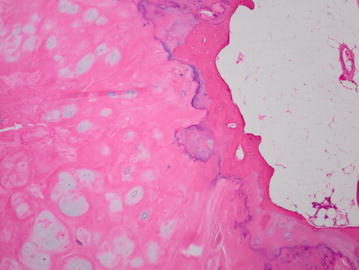
Tumor pathology. Microphotograph of the specimen showed mature trabecular bone and fatty marrow capped by cartilaginous tissue (×100, hematoxylin and eosin)

**Fig. 5:**
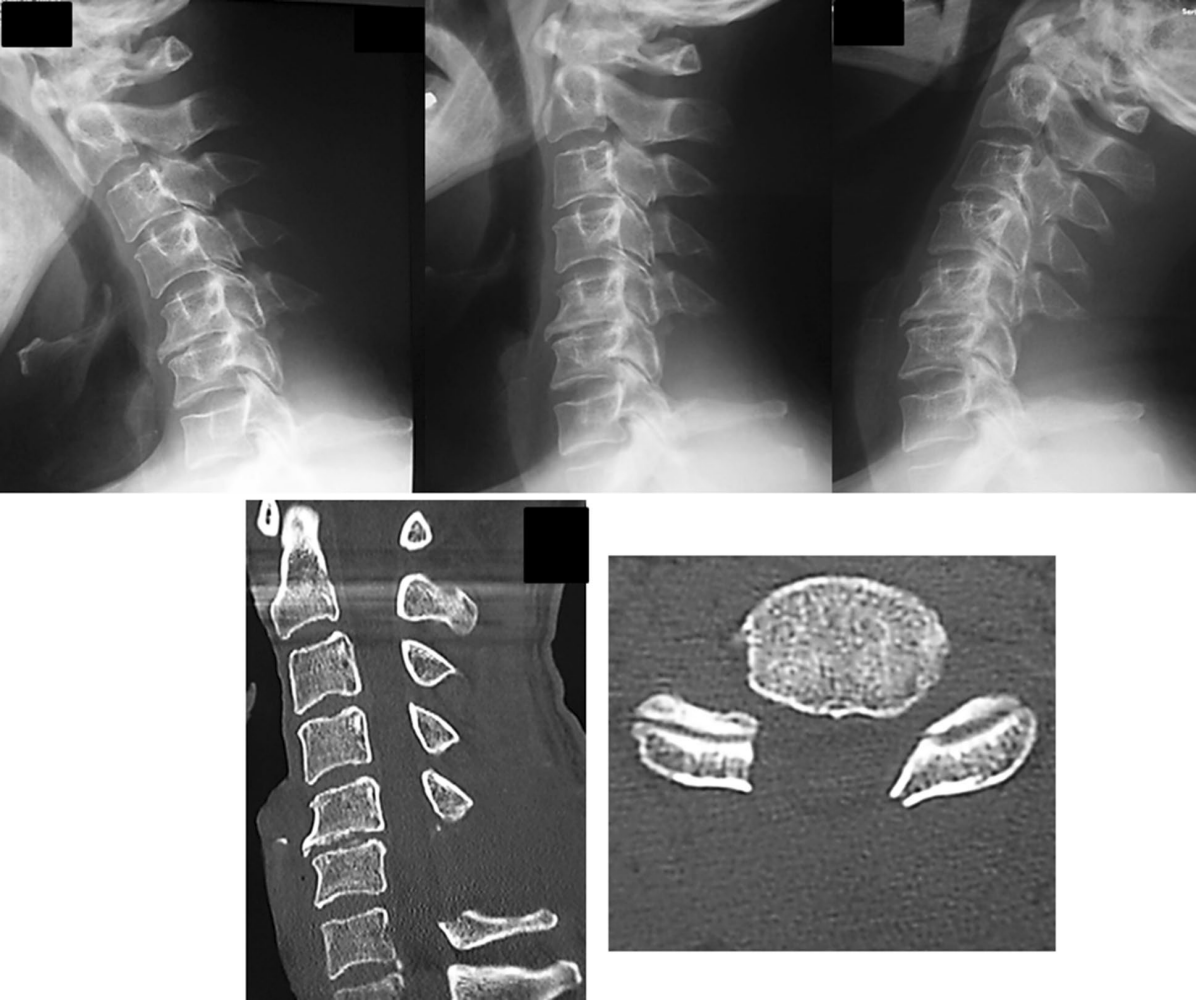
Postoperative plain radiograph and CT of the cervical spine. Post-operative radiograph of the cervical spine did not show each segmental instability. Moreover, post-operative CT scan showed complete resection of the tumor, and demonstrated that over 2/3 part of the facet was preserved

We diagnosed him with cervical myelopathy caused by the compression of the spinal cord due to an intra-canal tumor, and performed a C6 laminectomy and a C7 partial laminectomy with posterior approach. The tumor did not show the adhesion to the dural membrane, and *en bloc* resection of the tumor was performed. The resected tumor was about 2 cm in diameter, and its surface was covered with cartilaginous tissue (Fig. 3). The pathologic examination indicated mature trabecular bone with a cartilaginous cap, and we had a diagnosis of a benign osteochondroma (Fig. 4). On the next day after the surgery, the pain on the right upper extremity was improved, but the symptoms on the lower extremities remained. Therefore, we performed a decompression of the spinal canal at L3/4 level 1 month later. And then the symptoms improved clearly. He was discharged from our hospital at 6 weeks after his first operation. Post-operative radiograph of the cervical spine did not show each segmental instability. Also, post-operative CT scan showed the removal of the lesion, and demonstrated that over 2/3 part of the facet was preserved (Fig. 5). Recurrence was not observed at the time of examination 1 year after the first surgery.

## Discussion

Osteochondroma may develop as a solitary or multiple lesions which are known as hereditary multiple exostoses (HME). The incidence rate of HME is about 12 % of all osteochondromas (Gille et al. 2005). Particularly, the incidence of osteochondroma in the spine is reported to be very rare in the literature (Khosla et al. 1999). The spinal involvement of HME is about 7–9 % (Giudicissi-Filho et al. 2006), whereas that of solitary osteochondroma is about 1–4 % (Chooi et al. 2005). This tumor can occur in any vertebrae of the spine, but the cervical spine is commonly involved in about 50 % (Murphey et al. 2000). The most frequent site is the C2 vertebral bone, followed by C3 and C6 (Chatzidakis et al. 2007; Maheshwari et al. 2006; Schomacher et al. 2009). Osteochondroma typically occurs in young patients, but those of the spine usually occur during the second to third decades of life (Bess et al. 2005; Sakai et al. 2002). Sakai et al. suggested that degenerative changes of the vertebrae might contribute to the initiation of symptoms in the elderly. Some authors reported that both multiple and solitary osteochondromas are more likely to be appeared in the male than the female (Gille et al. 2005; Labram and Mohan 1996). However, Zaijun et al. (2013) reported that female patients showed symptoms more frequently than male patients in solitary osteochondroma of the spine [9:3].

Osteochondroma of extremities typically forms a pedunculated or a sessile mass adjacent to the bone marrow and the cortex (Albrecht et al. 1992). The plain radiological finding is usually useful for the diagnosis with the osteochondroma of the extremities. However, an osteochondroma of the spine is difficult to detect on only plain radiological findings, because the osseous structures of the spinal column are very complex (Yukawa et al. 2001). Most of the lesions typically arise from the lamina or pedicles, but can rarely arise from the vertebral body (Khosla et al. 1999). Lotfinia et al. (2010) reported plain radiological findings helped to identify lesions in 50 % of patients suffering from an osteochondoroma of the spine. Therefore both CT scan and MRI were recommended to detect the origin and size of the lesions with details. The CT scan can demonstrate the bony and cartilaginous components of the tumor, and is useful to clarify the zone of attachment of the tumor, contributing to preoperative planning (Sharma et al. 2002). The level and the extent of neural structure compression are best visualized on MRI. There are ossification of the ligamentum flavum (OLF) and bony spur as the lesions that we have to differentiate from osteochondroma. In East Asian countries, ossification of OLF is more common than in other countries. The prevalence of OLF was reported to be 3.8 % (whole spine) in China, and 36 % (thoracic only) in Japan (Guo et al. 2010; Mori et al. 2013). OLF is not commonly revealed at the cervical spine. It is possible to distinguish OLF from osteochondroma as the shape.

It was reported to be extremely rare that the osteochondroma of the spine compressed the spinal cord (Labram and Mohan 1996). The incidence of neurological symptoms including myelopathy and radiculopathy caused by the tumor was 0.5–1 % (Brastianos et al. 2005; Ratliff and Voorhies 2000). Rare osteochondroma arising anteriorly from the cervical vertebral body can compress the surrounding tissues, and cause dysphagia (Grivas et al. 2005; Wong et al. 2013), sleep apnea (Wang and Chou 2009), respiratory distress, vocal cord paralysis (Certo et al. 2014), and Horner syndrome (Zhao et al. 2007). Moreover, Certo et al. (2014) reported the correlation between tumor development and presence of diffuse idiopathic skeletal hyperostosis. Symptomatic lesion needed to be treated surgically, and good results of the total resection were reported (Albrecht et al. 1992; Brastianos et al. 2005). The therapeutic option for an asymptomatic lesion is controversial. Some authors reported that the surgical intervention should be recommended when the spinal cord was compressed by the tumor (Aldea et al. 2006; Giudicissi-Filho et al. 2006; Wen et al. 1989). Sudden death caused by odontoid osteochondroma was reported (Rose and Fekete 1964).

The risk of recurrence is approximately 2–4 % in osteochondroma of the spine (Bess et al. 2005; Gille et al. 2005). Pecker et al. (1980) reported a case of recurrence treated by curettage of the tumor lesion. Bess et al. (2005) described two cases of symptomatic recurrence after intralesional resection. The mean interval of the recurrence was 4.2 years. The malignancy usually originates from the cartilaginous cap (Lotfinia et al. 2010). Therefore, some authors recommend complete *en bloc* resection of the tumor (especially cartilaginous cap) should be done to reduce recurrence (Gille et al. 2005; Zaijun et al. 2013). Zaijun et al. (2013) reported two cases with recurrence had sarcomatous transformation. The incidence of malignant transformation is reported to be approximately 1 % in a solitary osteochondroma and 10 % in HME (Brastianos et al. 2005; Khosla et al. 1999). If the tumor originates from the facet, we may consider a stabilization of the spinal column to remove it completely (Albrecht et al. 1992; Yukawa et al. 2001). In our case, the stalilization was not necessary because over 2/3 part of the facet joint was preserved.

## Conclusions

We demonstrated the case of osteochondroma in the cervical spine that was treated with surgery. It is very important to perform a complete *en bloc* resection of the tumor (especially cartilaginous cap) to prevent the recurrence.

## Abbreviations

CT: computed tomography
MRI: magnetic resonance images
HME: hereditary multiple exostoses
